# Anesthesia Strategies for Pediatric Ophthalmic Patients: Systematic Review of Recent Advances and Updates

**DOI:** 10.1007/s40135-025-00339-4

**Published:** 2025-11-01

**Authors:** Abdulrahman Mamoon Allaf, Ta Chen Chang, Neil H. Masters

**Affiliations:** 1 Department of Ophthalmology, Bascom Palmer Eye Institute, University of Miami Miller School of Medicine, 900 NW 17th St – Room 541, Miami, FL 331236 USA; 2 Department of Anesthesiology, University of Miami Miller School of Medicine, Miami, FL USA

**Keywords:** Pediatric ophthalmology, Pediatric anesthesia, Emergence delirium, Examination under anesthesia, Retinopathy of prematurity, Retinoblastoma

## Abstract

**Purpose of review:**

This review summarizes recent advances in pediatric ophthalmic anesthesia, focusing on emergence agitation, examination under anesthesia (EUA), anesthesia strategies for retinopathy of prematurity (ROP), and care for children with developmental or medical comorbidities.

**Recent findings:**

Emerging evidence supports multimodal approaches to reduce emergence delirium, including sub-Tenon’s blocks with dexmedetomidine and intraoperative methadone. EUA protocols now emphasize streamlined exams under a single anesthetic, with some low-risk cases managed without intravenous access or general anesthesia. In ROP care, alternative strategies such as laryngeal mask airway use and bedside laser under sedation are being explored. Concerns over neurodevelopmental risks are also prompting efforts to limit cumulative anesthetic exposure through coordinated care.

**Summary:**

Pediatric ophthalmic anesthesia is evolving toward safer, more individualized approaches. Incorporating targeted techniques and minimizing exposure can improve outcomes and inform best practices in this vulnerable population.

## Introduction

Pediatric ophthalmic procedures often require general anesthesia to ensure immobilization and to minimize distress in a patient population with limited cooperation. As pediatric anesthetic care continues to evolve, there is increasing interest in optimizing anesthetic protocols to improve safety, efficiency, and patient experience. In addition to intraoperative considerations, emerging research has drawn attention to the potential neurodevelopmental effects of repeated anesthetic exposure in early childhood. This review highlights recent findings in pediatric ophthalmic anesthesia, with a focus on emergence agitation, examinations under anesthesia (EUA), anesthetic strategies in preterm infants with retinopathy of prematurity (ROP), and the perioperative management of children with developmental or medical comorbidities.

## Methods

An electronic search was conducted in PubMed for studies published between 2020 and 2025 using the search terms: "pediatric ophthalmology anesthesia." To ensure a comprehensive review, additional combinations were used, including (pediatric ophthalmology AND anesthesia), (strabismus OR cataract OR glaucoma OR retinoblastoma AND pediatric anesthesia), and (pediatric AND examination under anesthesia AND ophthalmology). The inclusion criteria were peer-reviewed studies that (1) examined anesthetic management in pediatric patients, (2) involved pediatric patients undergoing ophthalmic exams or surgery, and (3) provided practical details regarding perioperative protocols, outcomes, or management strategies relevant to clinical care. Studies focusing exclusively on adult populations, non-surgical interventions, or diagnostic imaging without anesthesia-related discussion were excluded.

This review was conducted following the Preferred Reporting Items for Systematic Reviews and Meta-Analyses (PRISMA) guidelines. Titles and abstracts were screened for relevance, followed by full-text review to assess eligibility. Studies that met the criteria were included in the final analysis. Reference management and data organization were performed using EndNote software (Clarivate, Berkeley, CA, USA).

## Results

**Fig. 1:**
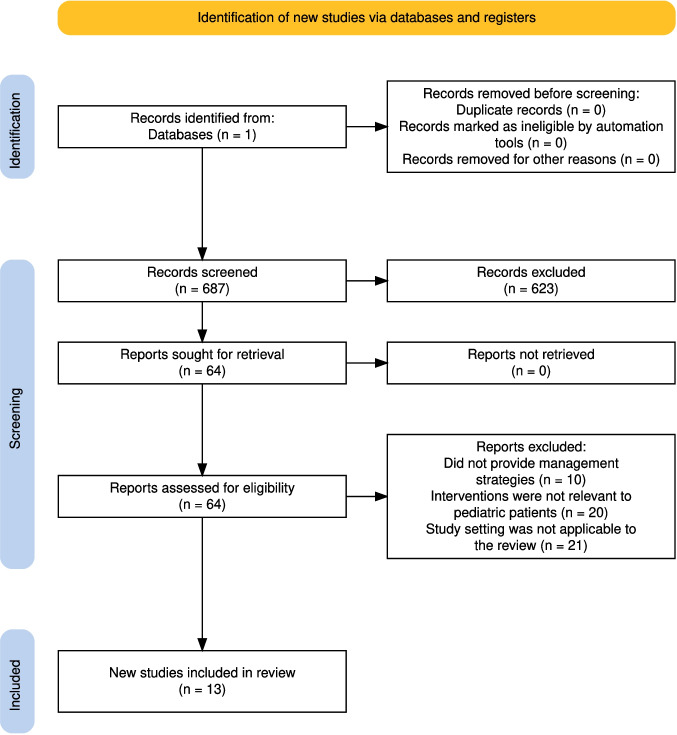
Preferred Reporting Items for Systematic Review and Meta-Analyses (PRISMA) diagram detailing the selection process for the studies included in this review

The search identified a total of **687** records, of which **64** were deemed relevant based on title and abstract screening. Following a full-text review, **51** studies were excluded, leaving **13** studies for inclusion in the final analysis (Fig. 1).

## Emergence Delirium and Agitation

Emergence delirium (ED) is a well-recognized complication following general anesthesia in pediatric patients, characterized by confusion, agitation, restlessness, crying, and inconsolability during the immediate postoperative period. The overall incidence of ED in children undergoing surgery is estimated to range between 10 and 30%, depending on anesthetic agents, age, and procedure type. However, higher rates have been consistently reported in certain surgeries, particularly in ophthalmology. A 2021 prospective study found the incidence of ED following pediatric strabismus surgery under sevoflurane anesthesia to be 22%, with younger age and poor preoperative sleep quality identified as significant risk factors [1].

Among the most promising strategies for reducing ED in pediatric ophthalmology is the use of regional anesthesia with adjuvant dexmedetomidine. In a randomized controlled trial of 80 children aged 2–8 years undergoing strabismus repair under sevoflurane anesthesia with laryngeal mask airway, patients receiving a sub-Tenon’s block with 0.5% bupivacaine combined with 0.5 µg/kg of dexmedetomidine exhibited significantly lower Pediatric Anesthesia Emergence Delirium (PAED) scores, reduced postoperative pain as measured by the Face, Legs, Activity, Cry, and Consolability (FLACC) scale, and fewer episodes of postoperative nausea and vomiting compared to those who received bupivacaine alone. Notably, the addition of dexmedetomidine did not affect emergence time, heart rate, blood pressure, or the incidence of oculocardiac reflex, indicating it is both safe and effective in this population [2]. These findings are consistent with the growing body of evidence supporting the use of dexmedetomidine via intravenous and intranasal routes for the prevention of postoperative ED in children, but represent a novel and targeted application in the setting of pediatric ophthalmic surgery [3].

Another agent showing benefit in ED prevention is methadone. In a single prospective study, pediatric patients undergoing ambulatory strabismus surgery were administered a single 0.15 mg/kg dose of methadone at the time of induction. Compared to historical controls, the methadone group had a 42% reduction in peak PAED scores and an 85% decrease in severe ED episodes. There were no significant differences in the post-anesthesia care unit (PACU) length of stay, respiratory events, or time to discharge, and the need for rescue analgesia was significantly lower in the methadone cohort. However, methadone’s long half-life raises concerns about postoperative respiratory depression and sedation, and it remains controversial among practitioners. [4].

Collectively, this evidence indicates a multimodal approach to minimizing ED in pediatric ophthalmic surgery. The addition of dexmedetomidine to regional blocks like the sub-Tenon’s technique and single-dose intraoperative methadone reduces the incidence and severity of ED. These strategies not only improve postoperative comfort and safety but may also shorten recovery times and enhance the overall surgical experience for patients, caregivers, and surgical teams. Future research should focus on defining optimal combinations, dosing thresholds, and age-specific protocols to further standardize care in this vulnerable population.

## Anesthetic Management of Infants with Retinopathy of Prematurity

Anesthetic planning for preterm infants undergoing treatment for ROP remains a complex and evolving aspect of pediatric ophthalmic care. These patients often present with unique challenges due to their low birth weight, immature physiology, and heightened sensitivity to respiratory and hemodynamic changes. While general anesthesia has traditionally been the most employed modality, particularly in operative settings where endotracheal intubation ensures airway security and procedural stability, recent evidence points to a broader range of viable strategies. These are increasingly being tailored to institutional resources and patient-specific factors.

In operative settings, the use of a laryngeal mask airway (LMA) has been explored as an alternative to endotracheal intubation. A recent retrospective cohort study evaluated 271 very low birth weight infants undergoing general anesthesia for ROP laser treatment and found that airway management technique significantly impacted postoperative outcomes. Specifically, infants managed with a LMA had a substantially lower need for postoperative mechanical ventilation (8%) compared to those managed with endotracheal tubes (ETT), where 74% required ventilation postoperatively. Even after controlling for differences in comorbidities, body weight, and anesthesia duration through multivariable regression and propensity score matching, the use of an LMA remained independently associated with a significantly lower likelihood of requiring mechanical ventilation. Notably, the average body weight in the LMA group was 2,110 g, which approaches the manufacturer’s minimum recommended threshold (2,000 g) and may have contributed to a 38% conversion rate from planned LMA placement to ETT due to improper seating. As such, these findings highlight the potential use of LMAs in carefully selected preterm infants to reduce postoperative respiratory complications and minimize neonatal intensive care unit (NICU) length of stay following ophthalmic surgery [5].

Sedation based protocols have gained traction as an alternative to general anesthesia, especially in centers performing laser therapy at the bedside within the NICU. These regimens often incorporate intravenous agents such as fentanyl, midazolam, or ketamine in combination with topical ocular anesthesia. Studies have shown that bedside laser under sedation can be both safe and effective, minimizing the need for intubation and reducing the stress associated with transport. However, this approach requires meticulous cardiorespiratory monitoring and the presence of personnel experienced in neonatal sedation to manage potential complications such as apnea or oxygen desaturation [6].

Taken together, these evolving strategies underscore the importance of individualized anesthetic planning in the management of preterm infants with ROP. While the use of LMAs and bedside sedation protocols may not be appropriate for all patients, emerging data suggest they could offer safe and effective alternatives to traditional general anesthesia in select cases. These approaches should be considered in the context of each infant’s clinical condition, institutional resources, and provider expertise. Further research is needed to better define patient selection criteria and ensure these evolving practices can be implemented safely across diverse care settings.

## Examination Under Anesthesia (EUA)

Examination under anesthesia (EUA) is essential when children are unable to cooperate during standard clinical examinations due to age, developmental delay, or anxiety. EUAs enable detailed evaluation of the anterior and posterior segments, precise intraocular pressure measurement, cycloplegic refraction, and diagnostic imaging. In many cases, they also allow the performance of minor therapeutic interventions such as suture removal, Meibomian gland and/or nasolacrimal duct probing, or intravitreal injections. When conducted comprehensively and efficiently, EUAs reduce diagnostic uncertainty, minimize repeated examinations, and streamline the course of treatment for ophthalmic conditions in children [7].

Preoperatively, a comprehensive anesthesia evaluation should include assessment of fasting status, airway anatomy, comorbidities, and previous anesthetic history. In syndromic children or those with developmental delay, particular attention should be given to potential airway challenges and medication sensitivities. A structured checklist should then be used to ensure all necessary ophthalmic instruments such as indirect ophthalmoscopes, refraction tools, tonometers, and mydriatic agents are prepared. A preinduction team briefing involving the ophthalmologist, anesthesiologist, nurses, and technicians is recommended to streamline the procedure and avoid missing key components. [7].

The traditional requirement for intravenous (IV) access during EUA has been reconsidered in recent literature. In a prospective observational study involving 82 healthy children or those with only mild systemic disease, aged 2 to 7 years and undergoing ophthalmologic EUA, those managed without IV access had significantly shorter induction and procedure times, experienced no intraoperative complications, and did not require emergency IV placement. The average EUA duration in this cohort was under 15 min, making mask induction without IV both safe and practical in select low-risk cases. Parental satisfaction was also notably higher in the no IV group, supporting mask induction without IV as an efficient and family-centered option for select low-risk cases [8].

A broader question of whether general anesthesia is necessary for all pediatric ophthalmic examinations is now being raised. In some cases, particularly in children with developmental delay, premedication with oral anxiolytics such as midazolam may provide a safe and effective alternative to facilitate the exam without escalation to general anesthesia. In a study of 50 ophthalmic examinations in 45 children with developmental delay, oral midazolam at a dose of 0.5 mg/kg (mean dose 11.8 mg) enabled successful completion of 98% of exams without the need for escalation to general anesthesia. The average time to examination was approximately 60 min, and there were no adverse events such as oxygen desaturation or the need for pharmacologic reversal. Although ancillary testing such as ultrasonography or fluorescein angiography was not performed, the authors noted that these modalities would have been feasible within the same exam session. These findings support the safe and effective use of midazolam in select patients with special needs who may otherwise be unable to tolerate clinic-based exams under topical anesthesia alone. The authors emphasized the importance of individualized dosing and close monitoring to balance efficacy and safety [9].

In addition to patients with developmental delay, certain genetic syndromes warrant special perioperative consideration during EUAs due to their associated systemic comorbidities. Children with Pierre Robin sequence, Treacher Collins, Goldenhar, Klippel-Feil, Apert, Crouzon, or any of the mucopolysaccaridoses often have craniofacial abnormalities that increase the risk of difficult mask ventilation and intubation, necessitating specialized airway equipment [10]. Those with trisomy 21 frequently present with hypotonia, macroglossia, and atlantoaxial instability, which complicate airway management and positioning; they also have a higher prevalence of congenital heart disease, necessitating cardiology clearance or cardiac anesthesia [11]. CHARGE syndrome is frequently associated with choanal atresia, cranial nerve dysfunction, and cardiac anomalies, making sedation and airway control more complex [12]. Similarly, DiGeorge syndrome (22q11.2 deletion) may involve hypocalcemia, immunodeficiency, and congenital heart disease, necessitating close multidisciplinary coordination [13]. These children frequently undergo EUAs or ophthalmic surgeries for strabismus, nasolacrimal duct obstruction, eyelid or orbital anomalies, colobomas, congenital cataracts, and optic nerve abnormalities. Careful coordination and contingency planning are essential to anticipate and manage potential complications.

Overall, these considerations support a modern, patient-centered approach to EUAs in pediatric ophthalmology. Recent evidence supports a more nuanced approach to patient selection and perioperative planning. In select low-risk cases, EUAs can be performed safely without intravenous access, and in some children with developmental delay, oral anxiolysis may reduce or eliminate the need for general anesthesia. These evolving strategies emphasize the importance of individualized care, interprofessional coordination, and thorough preoperative assessment to optimize safety, efficiency, and maximize clinical yield.

## Neurodevelopmental Considerations in Repeated Anesthetic Exposure

Children undergoing ophthalmic care may often require repeated exposure to general anesthesia early in life, particularly those with chronic conditions. This pattern of exposure has prompted growing interest in understanding the potential long-term effects of anesthesia on the developing brain. As evidence continues to evolve, it is essential to consider both the frequency and duration of anesthetic exposure when planning care for young children.

Frequent general anesthesia exposure is a significant concern in the management of pediatric patients with retinoblastoma. In one retrospective study of 43 children, the median cumulative anesthesia time was approximately 1,100 minutes per child, with the highest exposure seen in those undergoing intra-arterial chemotherapy and bilateral disease surveillance. Although individual procedures such as examination under anesthesia are relatively brief, their frequency contributes substantially to the total exposure [14]. This data aligns with growing concerns about the potential neurodevelopmental effects of repeated anesthesia in early childhood. Although brief anesthesia is generally considered safe, accumulating evidence suggests that multiple or prolonged exposures during critical windows of brain development may be linked to subtle impairments in cognition, attention, and executive function. This is especially relevant in pediatric ophthalmology, where children with chronic or bilateral conditions often undergo numerous procedures under general anesthesia before age five [15].

As a result, the long-term effects of pediatric anesthesia on neurodevelopment have become a focus of ongoing investigation. Large prospective studies such as the Pediatric Anesthesia Neurodevelopment Assessment (PANDA), Mayo Anesthesia Safety in Kids (MASK), and the General Anesthesia compared to Spinal anesthesia (GAS) trial have collectively shown that a single, short exposure to general anesthesia, typically under one hour, is not associated with measurable neurocognitive deficits in later childhood. This is especially reassuring in ophthalmic settings where brief procedures, like examinations under anesthesia, are common [16]. However, concerns persist regarding the effects of repeated or prolonged exposures during periods of rapid brain development. A systematic review and meta-analysis involving nearly 200,000 children found that multiple exposures to general anesthesia before age five were associated with a significantly increased risk of developing attention deficit hyperactivity disorder (ADHD), with a cumulative hazard ratio of 1.71. While these findings do not suggest causation, they underscore the importance of strategic planning in children requiring serial ophthalmic procedures [17].

As such, when managing patients who require multiple anesthesia exposures such as EUAs, scheduling should consider the child’s broader medical needs, especially in those with systemic comorbidities. Performing ophthalmic examinations during other planned procedures under the same anesthetic, such as neuroimaging or common surgical procedures, can reduce repeated exposures and improve safety without increasing anesthetic risk. This approach emphasizes the value of multidisciplinary coordination among ophthalmology, anesthesia, and other pediatric subspecialties to streamline care and enhance efficiency [18]. A summary of risks and management considerations across common pediatric ophthalmic anesthesia scenarios is provided in Table 1.

**Table 1:** Summary of risks and management considerations in pediatric ophthalmic anesthesia

Clinical scenario	Key risks	Management considerations	References
Emergence delirium/agitation	High incidence after strabismus; worsened by sevoflurane, young age, poor sleep	Sub-Tenon’s bupivacaine + dexmedetomidine (0.5 µg/kg) reduces PAED/FLACC scores [2]; IV/IN dexmedetomidine effective broadly [3]; single-dose methadone (0.15 mg/kg) decreases severe ED but requires monitoring [4]	[1–4]
Preterm infants with ROP	Respiratory vulnerability; apnea/desaturation risk; transport stress	In OR: LMA reduces postop ventilation compared with ETT (8% vs 74%) when ≥ 2 kg, though ~ 38% require conversion [5]. In NICU: sedation-based regimens (fentanyl, midazolam ± ketamine) with topical anesthesia can avoid intubation; requires continuous monitoring and experienced neonatal team [6]	[5, 6]
Low risk EUAs	IV start may prolong time, increase distress	Mask induction without IV safe in healthy children when EUA < 15 min; shorter induction; no intraop complications [8]	[7, 8]
EUAs in children with developmental delay or autism	Poor tolerance of awake exam; GA often required	Oral midazolam 0.5 mg/kg enables ~ 98% exam completion without GA, no adverse events [9]	[9]
Repeated EUAs (retinoblastoma, chronic conditions)	High cumulative exposure (median ~ 1100 min per child in retinoblastoma)	Consolidate procedures (EUA + MRI, injections, imaging) under single anesthetic; coordinate with other specialties	[14, 18]
Neurodevelopmental risk counseling	Parental anxiety over long-term effects	Reassure: single brief GA (< 1 h) not linked to deficits (PANDA, MASK, GAS) [12]; multiple/prolonged exposures may increase ADHD risk (HR 1.71) [13]; counsel honestly about uncertainties	[11–13]
Pierre Robin, Treacher Collins, Goldenhar, Klippel-Feil, Apert, Crouzon, etc	Craniofacial anomalies → difficult airway	Anticipate difficult intubation; prepare advanced airway equipment; maintain spontaneous ventilation until secure airway	[10]
Trisomy 21 (Down syndrome)	Macroglossia, hypotonia, atlanto-axial instability, CHD	Gentle airway handling; consider cervical spine precautions; obtain cardiology clearance as needed	[11]
CHARGE syndrome	Choanal atresia, cranial nerve dysfunction, CHD	Anticipate poor tolerance of sedation; early airway control; cardiac workup	[12]
22q11.2 deletion (DiGeorge)	Hypocalcemia, immunodeficiency, CHD	Correct electrolytes preop; strict aseptic technique; cardiac optimization	[13]

## Conclusions

Pediatric ophthalmic anesthesia continues to evolve, with increasing attention to perioperative safety, individualized care, and long-term outcomes. From mitigating emergence agitation through regional techniques to optimizing care for preterm infants with ROP and refining the approach to examinations under anesthesia, anesthetic management is becoming increasingly tailored to the specific needs of each patient. The evolving literature also highlights the importance of minimizing cumulative anesthetic exposure in children with chronic conditions and encouraging multidisciplinary coordination to consolidate care when feasible. As this field continues to advance, further research will be essential to define best practices across diverse patient populations and clinical settings.

## Key References

- El-Sherbiny SM, Kamal RA, Sadik N, Elshahat A. Effect of Dexmedetomidine in Sub-Tenon's Block on Emergence Agitation in Pediatric Strabismus Surgery under Sevoflurane Anesthesia. Anesth Essays Res. 2022;16(1):160–166. https://doi.org/10.4103/aer.aer_99_22.
  - Demonstrates the efficacy and safety of sub-Tenon’s dexmedetomidine in reducing emergence delirium in pediatric strabismus surgery. Represents a novel application of dexmedetomidine in ophthalmic regional anesthesia.
- Yang B, Lian C, Tian R, Chen Y, Tang S, Xiang H, et al. Twelve-year outcomes of bedside laser photocoagulation for severe retinopathy of prematurity. Front Pediatr. 2023;11:1,189,236. https://doi.org/10.3389/fped.2023.1189236.
  - Reports favorable long-term outcomes of bedside laser photocoagulation for ROP using sedation rather than general anesthesia, offering a potential alternative for select preterm infants.
- Sripadungkul D, Thanayongpibul R, Kasemsiri C, Wongwai P, Boonkamjad S, Litu D. Is Intravenous Access Necessary in Pediatric Patients Undergoing Ophthalmologic Examinations Under Anesthesia? A Prospective Observational Study. J Multidiscip Healthc. 2024;17:4637–4644. https://doi.org/10.2147/jmdh.S475544.
  - Supports selective omission of IV access during EUA in low-risk cases, though careful patient selection and safety measures remain essential.
- McInnis-Smith K, Chen K, Klanderman M, Abruzzo T, Ramasubramanian A. Quantity and duration of exposure to general anesthesia for pediatric patients with retinoblastoma. J AAPOS. 2022;26(6):313.e1–e5. https://doi.org/10.1016/j.jaapos.2022.07.013.
  - Quantifies anesthesia exposure in children with retinoblastoma and underscores the need for coordinated care to reduce cumulative anesthetic time and potential neurodevelopmental risks.

## Data Availability

No datasets were generated or analysed during the current study.
